# Examination of the Glycine Betaine-Dependent Methylotrophic Methanogenesis Pathway: Insights Into Anaerobic Quaternary Amine Methylotrophy

**DOI:** 10.3389/fmicb.2019.02572

**Published:** 2019-11-07

**Authors:** Adam J. Creighbaum, Tomislav Ticak, Shrameeta Shinde, Xin Wang, Donald J. Ferguson

**Affiliations:** ^1^Department of Microbiology, Miami University, Oxford, OH, United States; ^2^Department of Biological Sciences, University of Idaho, Moscow, ID, United States; ^3^Department of Biological Sciences, Miami University Regionals, Hamilton, OH, United States

**Keywords:** methanogenesis, quaternary amine, one-carbon, COG5598, glycine betaine

## Abstract

Recent studies indicate that environmentally abundant quaternary amines (QAs) are a primary source for methanogenesis, yet the catabolic enzymes are unknown. We hypothesized that the methanogenic archaeon *Methanolobus vulcani* B1d metabolizes glycine betaine (GB) through a corrinoid-dependent GB:coenzyme M (CoM) methyl transfer pathway. The draft genome sequence of *M. vulcani* B1d revealed a gene encoding a predicted non-pyrrolysine MttB homolog (MV8460) with high sequence similarity to the GB methyltransferase encoded by *Desulfitobacterium hafniense* Y51. MV8460 catalyzes GB-dependent methylation of free cob(I)alamin indicating it is an authentic MtgB enzyme. Proteomic analysis revealed that MV8460 and a corrinoid binding protein (MV8465) were highly abundant when *M. vulcani* B1d was grown on GB relative to growth on trimethylamine. The abundance of a corrinoid reductive activation enzyme (MV10335) and a methylcorrinoid:CoM methyltransferase (MV10360) were significantly higher in GB-grown B1d lysates compared to other homologs. The GB:CoM pathway was fully reconstituted *in vitro* using recombinant MV8460, MV8465, MV10335, and MV10360. Demonstration of the complete GB:CoM pathway expands the knowledge of direct QA-dependent methylotrophy and establishes a model to identify additional ecologically relevant anaerobic quaternary amine pathways.

## Introduction

Methane is a greenhouse gas with approximately 28 times greater potency than carbon dioxide (CO_2_) and is an important common fuel source (Auffret et al., 2017). To better understand the roles of methane in biogeochemical cycles and climate change, we must determine the routes of biological methane production and precursors for methanogenesis. The atmospheric concentration of methane is increasing (Yvon-Durocher et al., 2014) and this increase triggers feedback loops by releasing carbon from the permafrost in polar regions leading to increased methanogenesis (Olefeldt et al., 2013; Deng et al., 2017). Methanogenesis increases in response to elevated temperature, and methanogens demonstrate a larger temperature dependent flux than photosynthetic and other CO_2_ respiring organisms (Yvon-Durocher et al., 2014). Therefore, methane represents a larger percentage of increased atmospheric carbon emissions in response to warming (Yvon-Durocher et al., 2014). Our ability to predict future increases in methane relies upon expanding our knowledge of methanogenesis mechanisms, including those from higher order methylated ammonium compounds which are environmentally abundant (Mausz and Chen, 2019).

Methanogenesis proceeds through three known pathways: hydrogenotrophic, aceticlastic, and methylotrophic (Ferry, 2011). Methylotrophic methanogenesis has been well described from methanol, simple methylamines, and methylated sulfur compounds (Ferguson et al., 1996; Ferguson and Krzycki, 1997; Sauer et al., 1997; Sauer and Thauer, 1998, Ferguson et al., 2000; Tallant et al., 2000). In general, these pathways consist of a substrate-specific methyltransferase (Mt*x*B), a cognate corrinoid binding protein (Mt*x*C), and a secondary methylcorrinoid:coenzyme M methyltransferase (Mt*x*A). The *x* represents a substrate-specific designation for each protein (a = methanol, t = trimethylamine (TMA), b = dimethylamine (DMA), m = monomethylamine (MMA), and s = methylated sulfurs), except in the case of MtbA which can function in each of the three simple methylamine pathways (Ferguson et al., 1996; Ferguson and Krzycki, 1997). Additionally, an ATP-dependent activation enzyme such as RamA is intermittently required for reductive activation of the corrinoid binding protein Mt*x*C to the catalytically active Co(I) state (Ferguson et al., 2009). Once activated, the corrinoid binding protein can accept a methyl group from the substrate whose transfer is catalyzed by the Mt*x*B. The methylated Mt*x*C then acts as a substrate for the Mt*x*A for transfer of the methyl group to coenzyme M (CoM). The methyl-CoM then acts as a substrate for methyl-CoM methylreductase for production of methane (Ferry, 2011). During methylotrophic methanogenesis, reducing equivalents for reducing the methyl group on methyl-CoM to methane are typically gained by oxidizing one out of every four methyl groups to CO_2_, however, some methanogens use molecular hydrogen (H_2_) as an energy source to drive the pathway (Borrel et al., 2013; Enzmann et al., 2018).

A fortunate consequence of the study of methanogenesis from methylamines was the discovery of the genetically encoded amino acid *L*-pyrrolysine (Pyl) (Krzycki, 2005). It is suggested that Pyl evolved independently in each of the predicted active sites of MttB, MtbB, and MtmB as they are non-homologous enzymes, and removal of Pyl abolishes methanogenesis from each of the methylamine substrates (Krzycki, 2005). The TMA methyltransferase, MttB, is the namesake member of the widespread MttB COG5598 superfamily of enzymes, which has members spanning hundreds of species from both Bacteria and Archaea (Ticak et al., 2014). Interestingly, most members of COG5598 lack Pyl and are therefore likely not functional TMA methyltransferases. We recently showed that one non-Pyl (or Pyl-lacking) MttB homolog from *Desulfitobacterium hafniense* Y51 (DhMtgB) is a glycine betaine (GB) methyltransferase, suggesting a role of this family in breaking down higher order methylated ammonium compounds (Ticak et al., 2014).

Quaternary amines (QAs) are known to contribute to methanogenesis indirectly due to their breakdown by fermentative bacteria, which generates TMA (King, 1984). This is particularly important in marine environments where methylotrophic methanogens compete well, due to high sulfate concentrations in the sediments (Purdy et al., 2003). Recently, some methanogens have been reported to utilize QAs as a direct substrate (Tanaka, 1994; Watkins et al., 2012, 2014; Ticak et al., 2015). Several QAs, such as GB, choline, carnitine, and tetramethylammonium (QMA) are naturally produced and are utilized by organisms for functions as diverse as stabilizing osmotic pressure, biosynthesis, metabolizing fatty acids, or as toxic agents for defense (Rebouche and Seim, 1998; Craciun and Balskus, 2012; Ticak et al., 2014).

Despite all the work done on methylotrophic methanogenesis pathways, little is known about the pathways for QA-dependent methanogenesis. Methanogenesis from QMA was shown to proceed via the methylotrophic pathway using proteins apparently unique to its pathway (Asakawa et al., 1998). Unfortunately, the QMA-utilizing *Methanococcoides* strain NaT1 was lost and no genomic information was reported, leaving many unanswered questions regarding QA-dependent methanogenesis. Particularly, the sequence identity of the QMA:corrinoid methyltransferase (MtqB) compared to other analogous methyltransferases is unknown and whether MtqB contained Pyl is also unknown.

The GB-utilizing methanogen *Methanolobus vulcani* B1d (B1d) isolated by our laboratory (Ticak et al., 2015) is the focus of this study to address questions pertaining to methanogenesis from QAs. Here we report the pathway by which B1d performs direct GB-dependent methanogenesis. Consistent with our original published hypotheses (Ticak et al., 2015), we determined that methanogenesis from GB is initiated by a COG5598 methyltransferase and proceeds through a corrinoid-dependent pathway to methylate CoM. To our knowledge, this is the first reported intact methanogenic QA:CoM pathway that utilizes a Pyl-lacking MttB homolog and serves as a benchmark for further investigations into other QAs found naturally in the environment.

## Results

### Proteomic Analysis

**FIGURE 1:**
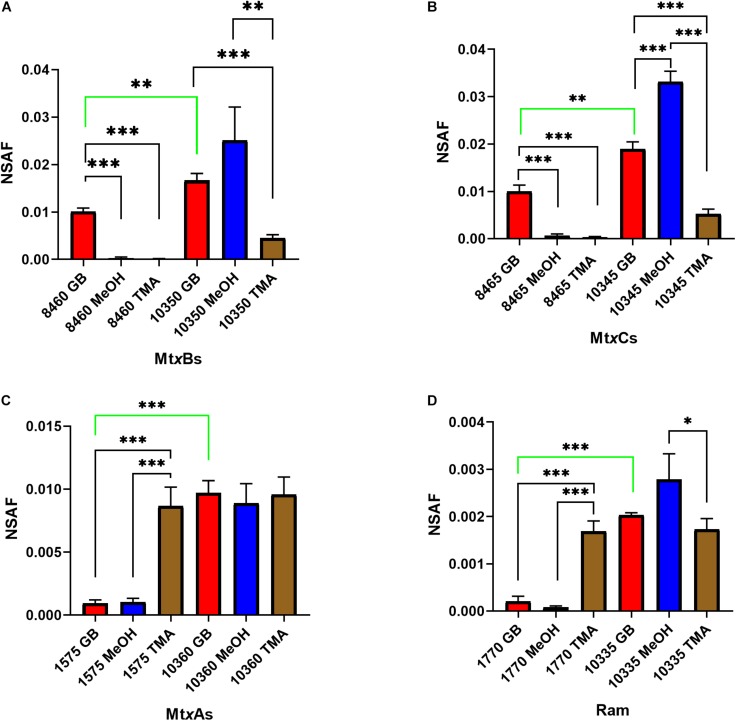
Proteomic analysis of likely candidate proteins for GB-dependent CoM methylation were analyzed: Mt*x*Bs **(A)**, Mt*x*Cs **(B)**, Mt*x*As **(C)**, and Ram **(D)** enzymes during B1d growth on GB, methanol, or TMA as the sole carbon source. Protein abundances were estimated by using the normalized spectral abundance factor (NSAF). Significant differences are indicated as follows when comparing individual protein levels between substrates: *p* ≤ 0.05 are shown by (^∗^), *p* ≤ 0.01 are shown by (^∗∗^), and *p* ≤ 0.001 are shown by (^∗∗∗^). The green bar indicates a significant difference between levels of analogous enzymes produced when grown on GB. Error bars represent standard deviations (*n* = 3).

**FIGURE 2:**
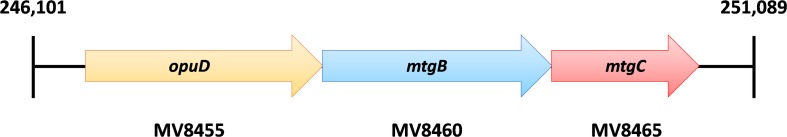
The genome of B1d encodes for a single homologous MttB that lacks pyrrolysine, *mtgB* (MV8460). Directly upstream of *mtgB* is the predicted GB transporter, *opuD*, and downstream is the cognate corrinoid binding protein, *mtgC*.

Significant gaps in knowledge remain regarding the pathways for methanogenesis from QAs. To begin addressing these gaps we analyzed the draft genome of B1d (Accession number VIAQ00000000) for genes of interest (Supplementary Table S1) and undertook a proteomic analysis of B1d during growth on GB, TMA, or methanol. GB-grown B1d compared to methanol- or TMA-grown B1d showed significant increases in MV8460 (Figure 1 and Supplementary Figure S1), a putative Pyl-lacking homolog of MttB from the COG5598 superfamily. The adjacently encoded putative cognate corrinoid binding protein (Figure 2), MV8465, showed significant increases in protein levels when growing in GB compared to methanol or TMA. The putative GB transporter, MV8455, encoded adjacent to MV8460 was present only during growth on GB (Supplementary Figure S1). MV10335, a RamM homolog required for activation of corrinoid binding proteins, was produced in significantly higher amounts during growth on methanol compared to TMA but levels on GB were not significantly different to methanol or TMA. MV10360, a MtaA homolog required for methylation of CoM, was produced at equal levels during growth on each substrate. However, compared to their homologs (MV1575 and MV1770) MV10360 and MV10335 were significantly higher during growth on GB. In addition to MV10335 and MV10360, MV10345 (MtaC) and MV10350 (MtaB) were present during growth on each substrate, suggesting the possibility of an available intact methanol:CoM pathway regardless of substrate.

### Phylogenetic Tree and Sequence Acquisition

**FIGURE 3:**
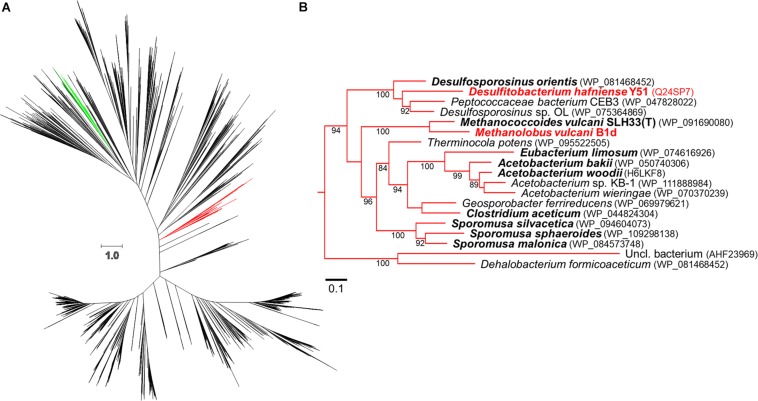
Approximate-maximum likelihood representation of the COG5598 MttB superfamily. The above phylogenetic tree **(A)** shows the proposed evolutionary relationship between both trimethylamine methyltransferases (dark green, archaeal; light green, bacterial) and GB methyltransferases (red). A portion of the GB clade is expanded in **(B)** with bootstrap values (greater than 80) being positioned at nodes. Bold names represent members previously reported to utilize GB while red names indicate biochemically demonstrated enzymes, accession numbers for the enzymes are in parentheses. The scale bars represent amino acid substitutions per site in both **(A**,**B)**.

We generated an updated COG5598 phylogenetic tree (Figure 3), from our prior analysis (Ticak et al., 2014). The WAG + CAT (Whelan and Goldman, 2001) tree provided the fewest bad-splits (17/2358) and the best log-likelihood (−1119437.408) with a Δ log-likelihood (7.948). The clade that contains the bona-fide MtgB from *D. hafniense* Y51 (DhMtgB) was analyzed for organisms reported to grow anaerobically with GB. The majority of the reported organisms capable of anaerobic GB-dependent growth from the clade are Clostridiales, with only two archaeal members present: *Methanococcoides vulcani* SLH33(T) and B1d (Muller et al., 1981; Möller et al., 1984; Dehning et al., 1989; Finster et al., 1997; Kuhner et al., 1997; Nielsen et al., 2006; Sattley and Madigan, 2007; Sikorski et al., 2010; L’Haridon et al., 2014; Ticak et al., 2014, 2015; Poehlein et al., 2015; Lechtenfeld et al., 2018).

### Structural Modeling of MV8460

**FIGURE 4:**
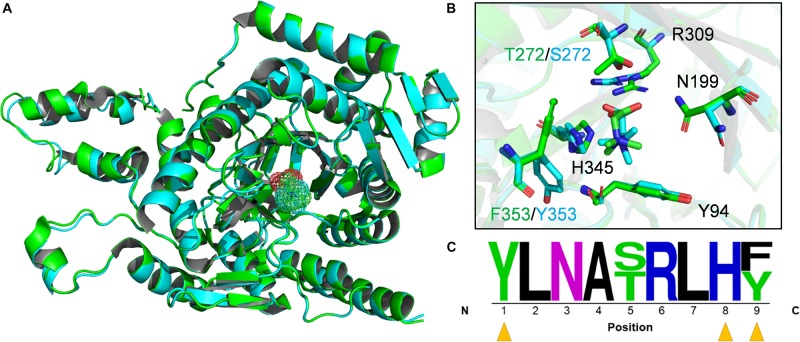
Active site predictions of DhMtgB and MV8460. **(A)** The models represent the aligned global structure of DhMtgB (green) docked to GB (green mesh) and homology model of MV8460 (cyan) docked to GB (cyan mesh). **(B)** The proposed interacting residues of both DhMtgB and MV8460 are shown within the TIM-barrel funnel which was highlighted with MetaPocket 2.0 (Huang, 2009). Black labeled residues indicate those shared between both structures while divergent amino acids are coded by either green (DhMtgB) or cyan (MV8460). **(C)** Active site logo generated after aligning both DhMtgB and MV8460 residues within 4–5 Å of the proposed docking site of GB were highlighted and aligned from N-terminus to C-terminus. Yellow arrows indicate aromatic residues which may interact in cation-π interaction with the methyl moiety of GB.

Based on the draft B1d genome, MV8460 is the sole Pyl-lacking MttB found in B1d, which is 65% identical and has 83% sequence similarity to DhMtgB. We therefore generated models of MV8460 using the apo-crystal structure of DhMtgB (PDB – 2QNE) as a template and compared the structures and predicted active sites of the two enzymes (Figure 4). The model of MV8460 generated using I-TASSER was the most accurate [C-score (2), TM-score (0.99 ± 0.04), and RMSD (2.9 ± 2.1 Å)] when compared to 2QNE. The structural motifs of DhMtgB and MV8460 are α/β TIM-barrel folds, much like other methyltransferase enzymes (Hao et al., 2002; Hagemeier et al., 2006). MetaPocket 2.0 (Huang, 2009) highlighted an eight β-sheet orientation in the center of the enzyme which generates a deep funnel that was indicated as a possible region for GB interaction. GB was docked at this location in DhMtgB with a predicted kCal/mol of −3.9 and MV8460 with a predicted kCal/mol of −3.7 and their global structures overlaid (Figures 4A,B).

The binding sites of the overlapped structures were analyzed for conserved or semi-conserved residues reported to bind GB and an active site logo was generated (Figure 4C). Various crystal structures of GB-binding enzymes; 1R9L (Schiefner et al., 2004), 6EYG, 3TMG (Li et al., 2015), 1SW2 (Li et al., 2015), 2B4L (Horn et al., 2006), 4MJW (Salvi et al., 2014), and 3L6H (Wolters et al., 2010); not related to the COG5598 superfamily, were used to validate the modeled GB ligand. The predicted binding site of GB in DhMtgB and MV8460 is most comparable to the GB-bound 4MJW crystal. The S101, H466, and N510 residues in 4MJW coordinate the carboxyl moiety of GB for cation-π interactions with the surrounding aromatics. This suggests that four predicted active site residues may interact with GB as they are conserved between DhMtgB and MV8460: Y94, N199, R309, and H345.

### MV8460 GB:cob(I)alamin Activity

**FIGURE 5:**
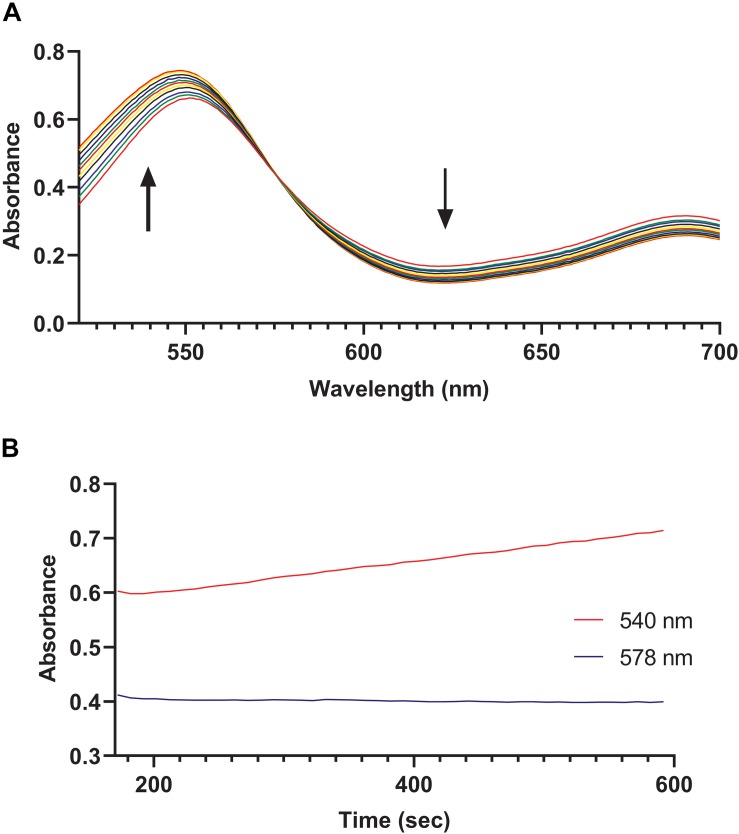
GB:cob(I)alamin methyl-transfer activity of MV8460. Over a period of 450 s, readings were taken every 10 s using an HP 8453 photodiode array spectrometer to monitor conversion of cob(I)alamin to methylcob(III)alamin. **(A)** Each spectrum represents a separate time point. The black bold arrows indicate that as the reaction progresses the absorbance increases at 540 nm while decreasing at 620 nm. Methylation of cob(I)alamin results in an increase at 540 nm and decrease at 620 nm. **(B)** No apparent changes at 578 nm with increases at 540 nm suggests direct conversion of cob(I)alamin to methylcob(III)alamin.

We hypothesized that the MV8460 was responsible for initiating methanogenesis from GB by catalyzing the corrinoid-dependent demethylation of the substrate, analogous to the function of DhMtgB (Ticak et al., 2014). We detected methylation of free cob(I)alamin by MV8460 using GB as the methyl donor, exhibited by an increase at 540 nm (Figure 5A). Cob(I)alamin and methylcob(III)alamin share an isosbestic point at 578 nm which is disrupted in the presence of cob(II)alamin (Kreft and Schink, 1993; Ticak et al., 2014). Inadvertent oxidation of cob(I)alamin can cause a false positive due to the formation of cob(II)alamin, causing increases at 540 and 578 nm (Kreft and Schink, 1993; Ticak et al., 2014). Absorbance at 578 nm remained unchanged throughout the assay, suggesting a direct conversion of cob(l)alamin to methylcob(III)alamin (Figure 5B). The specific activity of the recombinant MV8460 under the conditions tested was 0.21 μmol min^–1^ mg^–1^. Activity was not detected when choline, TMA, or QMA were used as methyl donor substrates.

### MV8465 Reduction by MV10335 Followed by Methylation of MV8465 by MV8460

**FIGURE 6:**
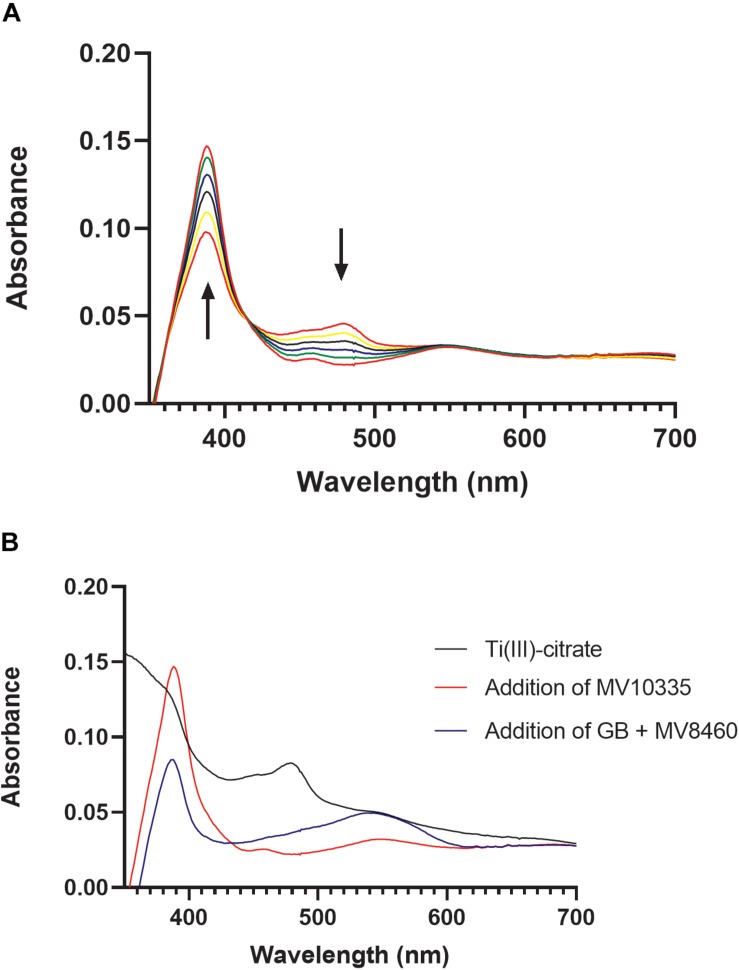
Reductive activation of MV8465 by MV10335. In order for methylotrophic methanogenesis pathways to function, the corrinoid binding protein must be in the active Co(I) state. MV10335 functions to reduce MV8465 to this active state. Ti(III)-citrate serves as an electron source for MV10335 but can also reduce MV8465 to Co(II), and therefore was added prior to the addition of MV10335. **(A)** Each spectrum is a time point with the black arrows indicating an increase absorbance at 378 nm and a decrease in absorbance at 478 nm, which is characteristic of reduction from Co(II) to Co(I). **(B)** MV8465 was initially reduced to Co(II) by Ti(III)-citrate to a stabilized spectrum (black line) and was then reduced to a Co(I) by the addition of MV10335 (red line). The major peak seen at 386 nm is characteristic of a Co(I) species. Addition of MV8460 and GB resulted in a decrease at 386 nm and an increase at 540 nm (blue line) indicating the formation of methylcob(III)alamin.

In *Methanosarcina barkeri*, reduction of the corrinoid binding proteins in the TMA, DMA, and MMA pathways relies on the RamA enzyme, and likewise, reduction of the corrinoid binding proteins in the methanol pathway relies on RamM (Ferguson et al., 2009). MV8465 displayed the characteristic UV-visible spectrum of a corrinoid binding protein (Figure 6A; Ferguson and Krzycki, 1997). We tested the ability of MV10335 to reduce the bound corrinoid of MV8465 from a Co(II) to a Co(I) state. MV8465 was partially reduced to the Co(II) form with excess Ti(III)-citrate to a stabilized absorbance spectrum, consistent with the corrinoid being in the Co(II) state (Kreft and Schink, 1993) (Figure 6B). The addition of MV10335 resulted in a significant increase at 386 nm, indicative of the Co(I) state. Following reduction of MV8465 by MV10335 we added MV8460 and GB as the methyl donor, which resulted in an observed decrease at 386 nm with a concomitant increase at 540 nm (Figure 6B).

### MV10360 Methylcob(III)alamin:CoM Activity

The penultimate step to methanogenesis is the methylation of CoM, and achievement of this step in the TMA degradation pathway is through methylcob(III)alamin:CoM methyltransferase (Mt*x*A) (Ferguson et al., 1996; Ferguson and Krzycki, 1997). CoM methylase activity of MV10360 was confirmed by monitoring change at 540 nm. The specific activity of the recombinant MV10360 under the conditions tested was 1.6 μmol min^–1^ mg^–1^. Cob(II)alamin is generated during methylcob(III)alamin:CoM methyl transfer, causing a decrease at 540 nm (Ferguson et al., 2011; Supplementary Figure S2).

### GB:CoM Reconstitution and Methanogenesis Assays

**FIGURE 7:**
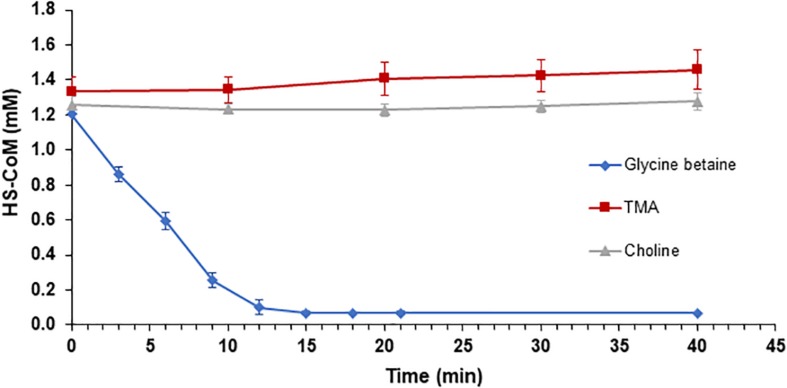
Reconstitution of GB:CoM activity *in vitro* with purified recombinant proteins. Loss of the free thiol group on CoM was monitored at 412 nm, using Ellman’s reagent. Addition of GB resulted in a significant decrease in the amount of HS-CoM that is detectable (blue diamond), indicating an intact GB:CoM methyl transfer pathway. Methylation of CoM was not detectable when TMA (red square) or choline (gray triangle) served as the methyl donors. The assays contained 10 μg of MV8460 and 5 μg each of the remaining proteins. Assays in which MV8460, MV8465, MV10335, or MV10360 were omitted lacked GB:CoM activity (data not shown). Error bars represent standard deviations (*n* = 3).

Following enzymatic confirmation of individual enzyme activities (MV8460, MV10335, and MV10360) predicted to be involved in the proposed GB:CoM pathway, we tested our hypothesis that GB-dependent methanogenesis occurs through a corrinoid-dependent methyl transfer pathway initiated via MV8460. We successfully reconstituted GB:CoM methyl transfer using highly purified recombinant proteins (Figure 7 and Supplementary Figure S6). MV8460, MV8465, MV10335, and MV10360 are each required for methylation of CoM. No CoM methylation was detected when using choline, QMA, or TMA as methyl donors.

To confirm that the activity we observed was not an artifact of recombinant proteins, we performed *in vitro* GB:CoM activity assays using crude lysates from B1d (Supplementary Figure S3). Extracts from GB-grown cells methylated CoM when GB was the methyl donor, at a rate of 0.27 umol min^–1^ mg^–1^, but not with TMA or methanol. Extracts from TMA-grown cells methylated CoM when TMA was the methyl donor, at a rate of 0.31 μmol min^–1^ mg^–1^, but not with GB, choline, or methanol. Surprisingly, CoM methylation was undetectable in extracts from methanol-grown cells using methanol, TMA, or GB as methyl donors, despite the extracts having been prepared identically to extracts of GB- or TMA-grown cells. The lower limit of detection for this assay was a loss of 0.3 mM free CoM over the course of a 40 min assay.

Extracts prepared from B1d cells grown on GB, TMA, or methanol lacked detectable methanol:CoM activity, we therefore tested for methanogenesis from prepared live cells, following previously established methods (Tallant and Krzycki, 1997). Washed cells grown on GB, TMA, or methanol showed rapid methane production when provided the same substrate, but a lag was detected if the cells were provided a different substrate (data not shown).

### MtaB Modeling

Due to the presence of MV10350 during growth on GB (Figure 1A and Supplementary Figure S1A), we modeled MV10350 to determine if GB could fit into the catalytic active site (Supplementary Figure S4). MV10350 shares 72.77% sequence identity and 99% query coverage with *M. barkeri* Fusaro MtaB (UniProtKB – Q46EH3). I-TASSER generated a homology model of MV10350 with a C-score (2), TM-score (0.99 ± 0.03), and RMSD (2.5 ± 1.9Å) to *M. barkeri* MtaB (PDB – 2I2X) (Hagemeier et al., 2006) (Supplementary Figure S4A). We used the proposed active site motif of MtaB (Supplementary Figure S4B) for predictive docking of methanol and GB to MV10350. The hydroxyl-group of methanol is located either near zinc, C219 or E312 in MV10350 (Hagemeier et al., 2006). Attempts to dock GB resulted in poor ligand positioning to cobalamin or steric hindrance due to zinc or the proposed potassium ion. Key residues involved with cation-π interactions for quaternary amine interactions are also lacking in MV10350 and therefore we could not accurately model GB into the active site of this enzyme.

## Discussion

**FIGURE 8:**
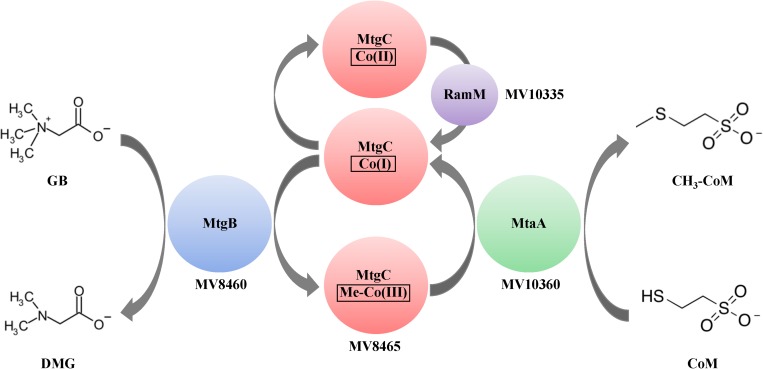
Proposed model of GB-dependent CoM methylation. Proteins are represented by colored circles/ovals and locations of genes that encode for them are also represented. The first methyltransferase, MtgB (blue), is a Pyl-lacking member of the COG5598 superfamily. MtgB catalyzes transfer of a methyl group from GB to the cognate corrinoid-binding protein, MtgC (red). The second methyltransferase, MtaA (green), catalyzes methyl transfer from Me-MtgC to CoM. The corrinoid reductive activation enzyme, RamM (purple), reduces the bound corrinoid of MtgC, when required. DMG is dimethylglycine.

Methanogenesis by B1d from GB as the sole carbon source results in approximately 0.75:1 stoichiometry of methane produced to GB consumed (Ticak et al., 2015). This suggested initial breakdown of GB is through a single demethylation reaction and not through the Stickland reaction that results in the formation of TMA and requires a betaine reductase (not present in the B1d draft genome) (Naumann et al., 1983). This work reports *in vitro* reconstitution of the methylotrophic GB:CoM pathway from B1d. Our work is consistent with published work on QMA-dependent methanogenesis (Asakawa et al., 1998), but expands it to include the identities of the genes encoding the enzymes of the pathway, the involvement of a Pyl-lacking COG5598 enzyme, and focuses on a likely more ecologically relevant QA. Our work is also consistent with a recently published study showing the involvement of another Pyl-lacking COG5598 enzyme and cognate corrinoid binding protein during methylotrophic growth of the gut bacterium *Eubacterium limosum* on the QA proline betaine (Picking et al., 2019). Given our results, we propose the following model for metabolism of GB from B1d (Figure 8).

Amongst the compounds tested, MV8460 appeared to only interact with GB, which is consistent with previous work on methylotrophic methyltransferases (Burke and Krzycki, 1995; Ferguson and Krzycki, 1997; Sauer et al., 1997; Sauer and Thauer, 1998; Ferguson et al., 2000; Tallant et al., 2000; Pritchett and Metcalf, 2005; Ticak et al., 2014). In-depth analysis of the biochemical interactions between MV8460 and MV8465 was beyond the scope of this work and therefore we did not determine if MV8460 could interact with another corrinoid binding protein from a different methylotrophic pathway. However, productive interaction of methylotrophic methyltransferases with non-cognate corrinoid binding proteins has never been reported, to our knowledge. Therefore, interaction of MV8460 with another corrinoid protein seems unlikely.

Until recently, the only characterized Pyl-lacking COG5598 homolog was from *D. hafniense* Y51 and it functions to demethylate GB (Ticak et al., 2014). The high similarity between MV8460 and DhMtgB coming from different domains of life piqued our interest to search for conserved amino acids and model DhMtgB and MV8460 for basic analysis of the catalytic pocket and docking of GB (Figure 4). Interestingly, in many of the crystal structures of enzymes which bind GB or other quaternary amines (Schiefner et al., 2004; Horn et al., 2006; Wolters et al., 2010; Salvi et al., 2014; Li et al., 2015), the methyl moiety is commonly flanked with aromatic compounds which are involved in cation-π and π-π stacking. The residues likely involved with either cation-π or π-π stacking are Y94 and F353/Y353 while the interaction of H345 is more complex. Given physiological pH and the pKa of histidine, H345 is likely protonated and acts as a hydrogen donor to the carboxyl moiety of GB much like 4MJW (Salvi et al., 2014). Additionally, given the distance (4.1 Å, DhMtgB; 4.6 Å, MV8460) and the positioning of H345 it is unlikely to interact in cation-π with the methyl moiety. It is more likely that H345 would be involved in π-π stacking with the nearby F353/Y353 if H345 was unprotonated. The position of GB within the predicted funnel would also involve R309 for positioning of the carboxylic moiety in both DhMtgB and MV8460 for helping coordinate the molecule and methyl group for catalytic attack by the Co(I) species from MV8465. This positioning of the methyl group for catalytic attack is analogous to what has been seen previously in MtaBC (Hagemeier et al., 2006).

The proteomic data revealed constitutive production of the proteins involved in methanol-dependent methanogenesis (Figure 1), at varying levels depending on the substrate. This suggests that methanol could be B1d’s most commonly available carbon source in the environment, which would make it advantageous for the organism to maintain production of the enzymes under all conditions. Interestingly, we could not detect methanol:CoM methyl transfer activity in any of our extracts. Therefore, we considered the possibility that the putative MtaB, MV10350, could be involved with GB metabolism. The MtaB from *M. barkeri* forms a tight complex with MtaC and supporting evidence suggests this complex is only active for methanol degradation (Sauer et al., 1997; Sauer and Thauer, 1998; Pritchett and Metcalf, 2005; Hagemeier et al., 2006). Additionally, our methanogenesis assays on methanol grown cells when they were fed GB showed no apparent activity within a 24 min assay (data not shown), indicating that the intact methanol pathway did not readily interact with GB. Therefore, we suggest that if interactions between MV10350 and GB were possible, it would not be active at biologically relevant levels. MV10350 and MV8460 are not homologous and our modeling data of MV10350 using the crystal structure of the *M. barkeri* MtaB (Hagemeier et al., 2006) as a guide, suggests that GB would not fit into the catalytic pocket of MV10350 (Supplementary Figure S4).

Given the homology between MtgB and MttB, we predicted the involvement of a RamA and MtbA in the GB pathway. However, given that MtaA from the methanol pathway from *M. barkeri* can function in the methanogenesis pathway for TMA (Ferguson et al., 1996; Ferguson and Krzycki, 1997), it is not surprising that MV10335 (RamM) and MV10360 (MtaA) are used during methanogenesis from GB in B1d. The genetic regulation of the methanol operon that encodes these two enzymes is not currently known and may be subject to future study. In available methanogen genomes, the first methyltransferase of methylotrophic pathways is consistently encoded adjacent to its cognate corrinoid binding partner (Galagan et al., 2002; Maeder et al., 2006; Webster et al., 2019). It is interesting to speculate that methylotrophic methanogenic metabolism of GB, or potentially other QAs, could be achieved with the acquisition of two genes that encode a methyltransferase and a cognate corrinoid binding partner, yet methanogenic pathways for other QAs as direct carbon sources have yet to be established. The methylotrophic methanogenic pathway for GB is now known, and in B1d it involves two proteins associated with a methanol methanogenic pathway. Many microorganisms in brackish or marine environments utilize GB transporters to internalize GB for osmoprotection or biosynthesis (Ticak et al., 2014, 2015). Therefore, in nutritionally depleted environments due to competition, an advantage may be gained by the acquisition of genes encoding the enzymes for GB methylotrophy. This suggests a role for horizontal gene transfer with these forms of metabolism and potentially explains the clustering of the Pyl-lacking COG5598 enzymes from the GB-utilizing B1d and SLH33(T) archaea in a bacterial dominated clade (Figure 3B).

## Materials and Methods

### Archaeal Strains, Media, and Growth

B1d was routinely cultivated under strict anaerobic conditions in brackish medium using either glycine betaine (GB) (80 mM), methanol (62.5 mM), or trimethylamine (TMA) (40 mM) (Ticak et al., 2015). *Methanosarcina acetivorans* WWM73 (WWM73) was a generous gift from Dr. William W. Metcalf (University of Illinois) and routinely cultivated using high-salt medium with methanol (62.5 mM) and acetate (40 mM) (Metcalf et al., 1997).

### Proteomics Analysis

Mid-log phase B1d grown on either GB (80 mM), methanol (62.5 mM), or trimethylamine (40 mM) was used for proteomics analysis following similarly to previously described methods (Ticak et al., 2015). Anoxically harvested cell pellets were resuspended in 12 mL of buffer (50 mM Tris–HCl, 10 mM CaCl_2_, 0.1% *n*-Dodecyl β-D-maltoside, pH 7.6), followed by cell lysis by French press, as described below. Cell lysates were centrifuged at 40,000 × *g* for 45 min at 4°C and the supernatants were collected and used for proteomic analysis. Protein concentrations of the supernatants were measured using the Bradford assay (Bradford, 1976) (Thermo Scientific).

For each sample, 100 μg of total protein was denatured in 8 M urea supplemented with 5 mM dithiothreitol. The protein solution was diluted to 2 M urea using the same Tris buffer, followed by a 1:100 w/w Trypsin Gold (Promega) protein digestion at 37°C for 18 h. Digested peptides were desalted using Sep-Pak C18 columns following the manufacturer’s protocol (Waters Corporation), followed by peptide fractionation using a Pierce High pH Reverse-Phase Peptide Fractionation Kit (Thermo Scientific). Eight peptide fractions of each sample were separated using a capillary C18 column on an EASY-nLC 1000 liquid chromatograph coupled to a Thermo LTQ Orbitrap XL mass spectrometer for MS analysis. The peptides were scanned in the range of 350–1800 m/z at a resolution of 30,000 operating in the data-dependent mode. For each scan, the 12 most abundant peaks were selected and subjected to MS/MS analysis by collision induced dissociation fragmentation. The peptide identities were searched against a B1d database (Genbank accession number VIAQ00000000) using pipeline programs integrated in PatternLab for Proteomics (version 4.1.0.17) and normalized spectral abundance factor (NSAF) was used to compare protein abundances. Raw data and the searched sqt files have been deposited to the MassIVE repository with the identifier MSV000084013.

### Cloning and Expression Vectors

B1d genomic DNA (gDNA) was extracted using phenol-chloroform (Sambrook et al., 1989) and the gene encoding MV8460 was amplified using primers shown in Supplementary Table S2. The product was digested with *Sac*II and *Xho*I (NEB) and treated with calf-intestinal alkaline phosphatase (NEB) before ligation into pASK-IBA43(+) (IBA Life Sciences) using T4-Ligase (NEB) to yield pASK_MV8460. The construct was maintained in *Escherichia coli* DH5α. The cloning of pASK_MV10335 followed similarly to pASK_MV8460.

The expression vector pET28(+) (Addgene) was modified to have a larger multiple cloning site. A DNA fragment containing an *E. coli* BL21(DE3) codon optimized version of the gene encoding MV10360 was purchased from Genscript in pUC57 and contained flanking restriction sites (Supplementary Figure S5). The fragment was removed from pUC57 via restriction digest with *Nco*I and *Bam*HI (NEB) and ligated into pET28(+) yielding pET28_MV10360_Opt. The optimized MV10360 gene was removed from pET28_MV10360_Opt using *Dra*I and *Pml*I (NEB) and the vector re-ligated, resulting in pETAC17a. The optimized MV10360 fragment was solely used as a tool to generate pETAC17a, however, the optimized gene was not used in our experiments. The gene encoding MV10360 was amplified from B1d gDNA before cloning into pETAC17a yielding pETAC_MV10360.

Cloning of MV8465 into pDL05c followed established methods (Longstaff et al., 2007) using the MV8465 + AsisI F and MV8465 + Tev R primers (Supplementary Table S2). A second amplification using MV8465 + AsisI F and Tev + His R added a 3′ hexahistidine tag. The fragment was digested with *Xho*I and *Sac*II and ligated into pDL03c yielding pDLAC03_MV8465 before cloning into pDL05c resulting in pDLAC05_MV8465 and was maintained in *E. coli* EC100 cells. Transformation into WWM73 followed previously established methods (Ladapo and Whitman, 1990; Metcalf et al., 1997).

### Production of Recombinant Proteins

Production of MV8460 followed similarly to established methods (Ticak et al., 2014). Anhydrous tetracycline (1 mg/L) was used to induce protein production. *E. coli* ArcticExpress (Agilent) was used to produce MV10360, following manufacturer’s protocol with modifications. Following the initial growth period prior to induction, cells were chilled at 4°C for 3 h. Cells were induced with 1 mM isopropyl β-D-1-thiogalactopyranoside (IPTG) and incubated at 10°C shaking at 125 RPM for 24 h. All cells were harvested by centrifugation at 7,500 × *g* for 15 min at 4°C and cell pellet stored at −80°C.

Production of MV10335 was done under anoxic conditions using *E. coli* SG13009, a gift from Dr. Joseph Krzycki (The Ohio State University). Cells were grown in anoxic LB supplemented with sodium phosphate (44 mM) prior to autoclaving and glucose (80 mM) and fumarate (80 mM) after autoclaving. The medium was bubbled with pure N_2_ for 30 min, after sterilization, then the flask was quickly stoppered and secured with copper wire. Additionally, 160 mL serum bottles were prepared containing 42 mL of the amended LB medium and made anoxic by stoppering the top and flushed and evacuated using N_2_, then sterilized by autoclaving and amended with glucose and fumarate. Starter cultures (50 mL) were grown statically overnight at 34°C. Starter cultures were transferred to the amended LB and grown at 34°C shaking at 125 RPM to an OD_600_ between 0.3 and 0.4 and then supplemented with cysteine (5 mM) and ferrous ammonium chloride (0.1 mM). Production was induced with IPTG (1 mM) followed by a 6 h incubation at 34°C shaking at 125 RPM. Cysteine and ferrous ammonium chloride were added again after 6 h incubation and the culture was further incubated for 2 h. Cells were harvested anoxically, as described above.

MV8465 production was done in WWM73 (Longstaff et al., 2007). Cells producing MV8465 were grown in 1-L of high-salt mineral media. The medium mixes A and B were prepared and autoclaved separately, mixed, and then amended with methanol (62.5 mM), acetate (40 mM), ampicillin (100 μg/L), and puromycin (2 μg/L). The medium was supplemented anoxically with filter sterilized: KH_2_PO_4_ (5 mM), cysteine-HCl (2.8 mM), ammonium chloride (19 mM), and Na_2_S (0.4 mM). A 5% (v/v) inoculum of WWM73 pDL05cMV8465 was grown statically at 37°C to OD_600_ ∼0.5 and transferred to the 1-L of high-salt media. The culture was grown statically at 37°C to stationary phase with periodic venting. Cells were harvested as described above.

### Protein Purification

Purification of His-tagged MV8460, MV10360, and MV8465 was done using established methods (Ticak et al., 2014), with additional steps. All proteins were partially purified anoxically using a HisTrap^TM^ HP using an ÄKTA Prime Plus (GE Healthcare). Lysates were loaded onto 1 mL columns and equilibrated with anoxic 95% Buffer A and 5% Buffer B (Ticak et al., 2014) for 10 column volumes and subjected to a 50-mL linear gradient from 5% to 100% Buffer B at 1 mL/min. MV8460, MV8465, and MV10360 each eluted as single peaks at 80–175, 85–145, and 65–145 mM imidazole, respectively. Protein peaks were pooled and diluted 1:5 with Buffer C (20 mM MOPS, 0 M NaCl, pH 7.8). Samples were loaded onto 1-mL Bio-Scale^TM^ Mini UNOsphere^TM^ Q cartridges (BIO-RAD) and equilibrated with 10 column volumes of Buffer C followed by a 0 to 70% Buffer D (20 mM MOPS, 1 M NaCl, pH 7.8) linear gradient. MV8460, MV8465, and MV10360 each eluted as single peaks at 330-420 mM, 240-430 mM, and 300-500 mM NaCl, respectively. MV8465 was diluted 1:5 with Buffer E (5 mM sodium phosphate, pH 7.0), loaded onto a 5-mL Bio-Scale Mini CHT Type I Cartridge (BIO-RAD), and equilibrated with 5 column volumes of Buffer E followed by a 0% to 100% Buffer F (500 mM sodium phosphate, pH 7.0) linear gradient following the manufacturer’s protocol. MV8465 eluted from the CHT column in a single peak at 185–210 mM phosphate. Protein purity was assessed via SDS-PAGE (BIO-RAD) followed by Coomassie blue staining (Supplementary Figure S6). Protein concentrations were determined by the Bradford protein assay (Bradford, 1976) and frozen at −20°C. Proteins were used within 6 months of freezing.

Materials needed for MV10335 purification were made anoxic 3 days prior to use. The cell pellet was resuspended in anoxic Buffer A and lysed via an anoxically adapted French press cell at 20,000 PSI. The anoxic lysate was spun at 250,000 × *g* at 4°C for 1.5 h, and then passed through a 0.22 μm syringe filter. MV10335 was purified as described above. MV10335 eluted as a single peak at 77–175 mM imidazole and as a single peak at 280–420 mM NaCl. Fractions were filtered into an anoxic Wheaton serum bottle. MV10335 degrades upon freeze-thaw cycles and therefore was stored at 4°C and used within 5 days after purification.

### MV8460 and MV10360 Methyltransferase Activity

Assays monitoring the activity of MV8460 followed described methods measuring the change at 540 and 578 nm at 37°C (Ticak et al., 2014). The reaction contained 50 μg (950 pmol) MV8460 and was initiated by addition of a methyl donor (50 mM). Measurements were taken every 10 s for 450 s. MV10360 activity assays followed described methods (Ferguson et al., 2011). Methylcob(III)alamin (595 μL) and CoM (5 μL) were added to an anoxic cuvette and incubated at 37°C for 10 min. The reaction was initiated by addition of 50 μg MV10360 and monitored until no detectable change at 540 nm.

### Reductive Activation of MV8465 by MV10335 Followed by Methylation of MV8465 by MV8460

Monitoring reduction of MV8465 followed described methods described (Ferguson et al., 2009). Reaction mixtures were assembled in sealed anoxic 0.2 cm cuvettes containing 12.5 mM ATP, 25 mM MgCl_2_, 4 mM Ti(III)-citrate, MV8465 (800 μg/ml), and 50 mM MOPS, pH 7.2. Reactions were incubated at 37°C monitoring change at 378 nm until the spectrum stabilized. MV10335 (216.5 μg/ml) was added and the 400 uL reaction was incubated for 45 min monitoring the change at 378 and 475 nm every 2 min. Upon stabilization of the spectrum, 3.75 μl each of MV8460 (144 μg/ml) and GB (18.75 mM) were added and the cuvette incubated for 30 min taking measurements every 2 min.

### *In vitro* Reconstitution of GB:CoM Methyl Transfer Pathway

Testing for functionality of individual proteins that were used for *in vitro* reconstitution of the GB:CoM pathway followed similarly to established methods and are summarized above and in Supplementary Material. All assays were performed as previously described (Ferguson et al., 2009, Ferguson et al., 2011; Ticak et al., 2014), with minor modifications. A 5x reaction mixture containing CoM (15 mM), ATP (62.5 mM), and MgCl_2_ (125 mM) was prepared in 50 mM MOPS, pH 7.2. The assay was performed in a stoppered anoxic cuvette with a final reaction volume of 250 μL. Ti(III)-citrate (Seefeldt and Ensign, 1994; Ferguson et al., 2011) amended MOPS was added followed by MV8460 (10 μg/190 pmol), MV10360 (5 μg/139 pmol), MV8465 (5 μg/174 pmol), MV10335 (5 μg/85 pmol), and the reaction mixture. Ten μL of Ti(III)-citrate (∼4 mM) was added as a source of reducing potential and the cuvette incubated at 37°C for 45 min. The reaction was initiated by adding 10 μL of the methyl donor (16 mM). Samples were taken every 4 min for 24 min and mixed with Ellman’s reagent (Ellman, 1958) in a 96-well round bottom plate and measured at 412 nm using a MolDev FilterMax F5 plate reader. The lower limit of detection for free CoM with Ellman’s reagent, in our hands, is a loss of 0.3 mM free CoM over the course of a 40 min assay.

### *Methanolobus vulcani* B1d Substrate Dependent CoM Methylation in Extracts

B1d cells were grown either on GB, methanol, or TMA and anoxically harvested during mid-log phase (Ticak et al., 2015). Cells were resuspended with anoxic MOPS (50 mM), pH 7.2 and anoxically lysed, as described above. Lysed cells were centrifuged, and lysates were filtered into sterilized anoxic serum bottles, as described above, and headspace exchanged with H_2_. The assay followed similarly to previously established methods (Ferguson et al., 2011). Ti(III)-citrate (∼4 mM) and a headspace of H_2_ were both required for substrate demethylation activity.

### Sequence Acquisition and Phylogenetic Construction of MttB Superfamily

The DhMtgB amino acid (aa) sequence (UniProtKB–Q24SP7) was used as a query with PSI-BLAST (Altschul et al., 1997) with a 1E^–10^ cutoff, for the non-redundant protein sequence (nr) database. Similarly, the MttB of *M. barkeri* (UniProtKB – O93658) was used to acquire Pyl-encoding MttB sequences via tBLASTn (Gertz et al., 2006). Sequences varying more than one standard deviation from the mean aa length and those with identities less than 30% of the query were removed. The remaining 5517 sequences were filtered using CD-Hit (Li and Godzig, 2006) to remove those with greater than 90% identity providing a final dataset of 2356 sequences averaging 504 aa. MV8460 and MttB10 (UniProtKB – H6LKF8) were added to the dataset from B1d and *Acetobacterium woodii*, respectively. The dataset was aligned with MUSCLE (Edgar, 2004) using default settings. Phylogenetic analysis was performed using approximately maximum-likelihood with FastTree 2 (Price et al., 2010), using JTT + CAT (Jones et al., 1992), WAG + CAT (Whelan and Goldman, 2001), and LG + CAT (Le and Gascuel, 2008), with and without Gamma distribution. Phylogenetic trees were generated using the interactive Tree of Life (iTOL) (Letunic and Bork, 2016).

### Homology Modeling Prediction of MV8460 and Molecular Docking of Glycine Betaine

The apo-structure of DhMtgB chain A (PDB – 2QNE) was used as a template to generate two models of MV8460 using both MODELLER (Webb and Sali, 2017) and I-TASSER (Zhang, 2008) for comparison. MetaPocket 2.0 (Huang, 2009) was used to predict possible binding site(s) for GB in DhMtgB and MV8460. Probably binding pockets and molecular docking predictions were performed using AutoDockTools and AutoDockVina, respectively (Trott and Olson, 2010).

Models with GB docked in the active site were visualized with PyMol v2.3. Aligned models were inspected for residues within 4–5 Å from GB to identify proposed active site signatures, generated with WebLogo3 (Sharma et al., 2012).

## Data Availability Statement

The datasets generated for this study can be accessed from the Genbank accession number VIAQ00000000, MassIVE repository identifier MSV000084013.

## Author Contributions

AC, TT, XW, and DF were involved in the design, performance, analysis, and interpretation of the experiments and wrote the manuscript. SS was involved in performance of the experiments.

## Conflict of Interest

The authors declare that the research was conducted in the absence of any commercial or financial relationships that could be construed as a potential conflict of interest.
