# Supplementary material for: Examination of the Glycine Betaine-Dependent Methylotrophic Methanogenesis Pathway: Insights Into Anaerobic Quaternary Amine Methylotrophy
Source: Front Microbiol. 2019 Nov 7;10:2572. doi: 10.3389/fmicb.2019.02572 (PMC6855144; doi:10.3389/fmicb.2019.02572)
Supplement: Supplementary file 1 [file Data_Sheet_1.docx]

**Supplementary Information**

**Homology modeling prediction of MV10345 and molecular docking of methanol and glycine betaine.**

The predicted methanol methyltransferase, MV10345, amino acid sequence was threaded using I-TASSER (Zhang, 2008) to the methanol methyltransferase portion of the methanol:cobalamin methyltransferase complex, MtaBC (PDB - 2I2X) (Hagemeier, et al. 2006). The predicted zinc-bound, and cobalamin-bound forms of MV10350 were aligned to 2I2X, chain A with the cognate corrinoid ligand of MtaC, zinc ions, and predicted potassium ion. The proposed active site of the *Methanosarcina barkeri* MtaB (Hagemeier, et al. 2006) was used to guide the docking studies with AutoDockVina (Trott and Olson, 2010) in MV10345. All structures were visualized with PyMol v2.3, both separately, and aligned with the *M. barkeri* MtaB.

**Methanogenesis assays**

Methanogenesis assays were performed similarly as previously described (Tallant and Krzycki, 1997). Briefly, B1d was grown on either GB, TMA, or methanol to mid-log phase and then chilled for 30 min. The cells were centrifuged at 8,500 x *g* for 20 min at 4°C in an anoxic environment. Cells were washed with ice-cold 50 mM MOPS buffer, pH 7.2, amended with NaCl (250 mM) and centrifuged again. The cells were resuspended in 4 mL of the same buffer and evenly divided into four 13.1 mL anoxic-Wheaton serum bottles. To the bottles, either GB, TMA, or methanol was added (25 mM) and incubated at 37°C shaking at 125 rpm. Methane was sampled every 6 min and ran at 5 mL/min through a TG-BOND Q 30 mm x 0.53 mm x 20 µm column into a Flame Ionization Detector (FID) that was attached to a Trace 1300 Gas Chromatograph Split/Splitless Injector instrument (Thermo Scientific).

**Supplemental Figures**


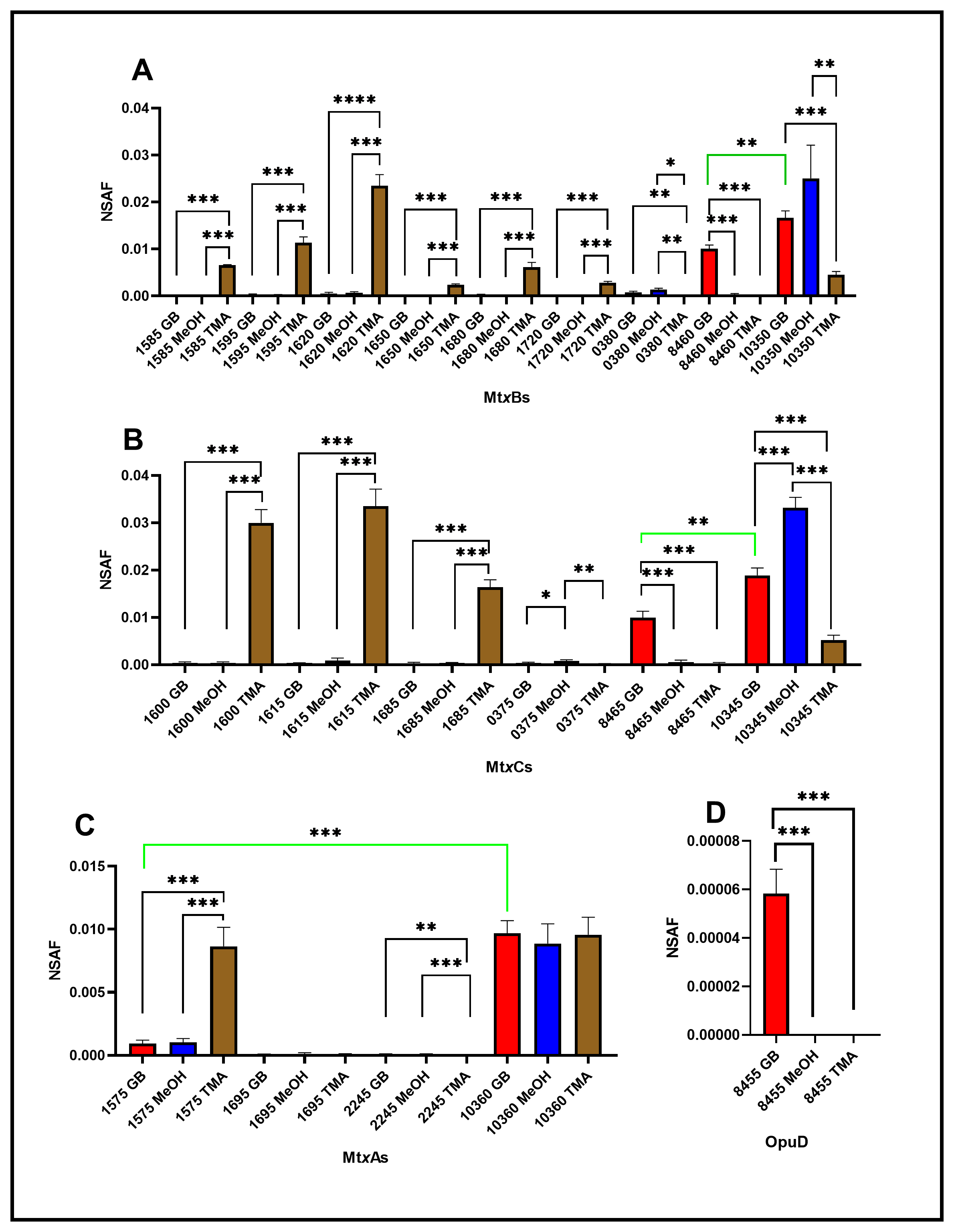


**Figure S1**. Proteomic analysis of likely candidate proteins for GB-dependent CoM methylation were analyzed: (**A**) Mt*x*Bs, (**B**) Mt*x*Cs, (**C**) Mt*x*As, and (**D**) OpuD (MV8455) during B1d growth on GB, methanol, or TMA as the sole carbon source. Protein abundances were estimated by using the normalized spectral abundance factor (NSAF). Significant differences, based on standard deviations (*n* = 3), are indicated as follows when comparing individual protein levels between substrates: *p* ≤ 0.05 are shown by (*), *p* ≤ 0.01 are shown by (**), and *p* ≤ 0.001 are shown by (***). The green bar indicates a significant difference between levels of analogous enzymes produced when grown on GB.


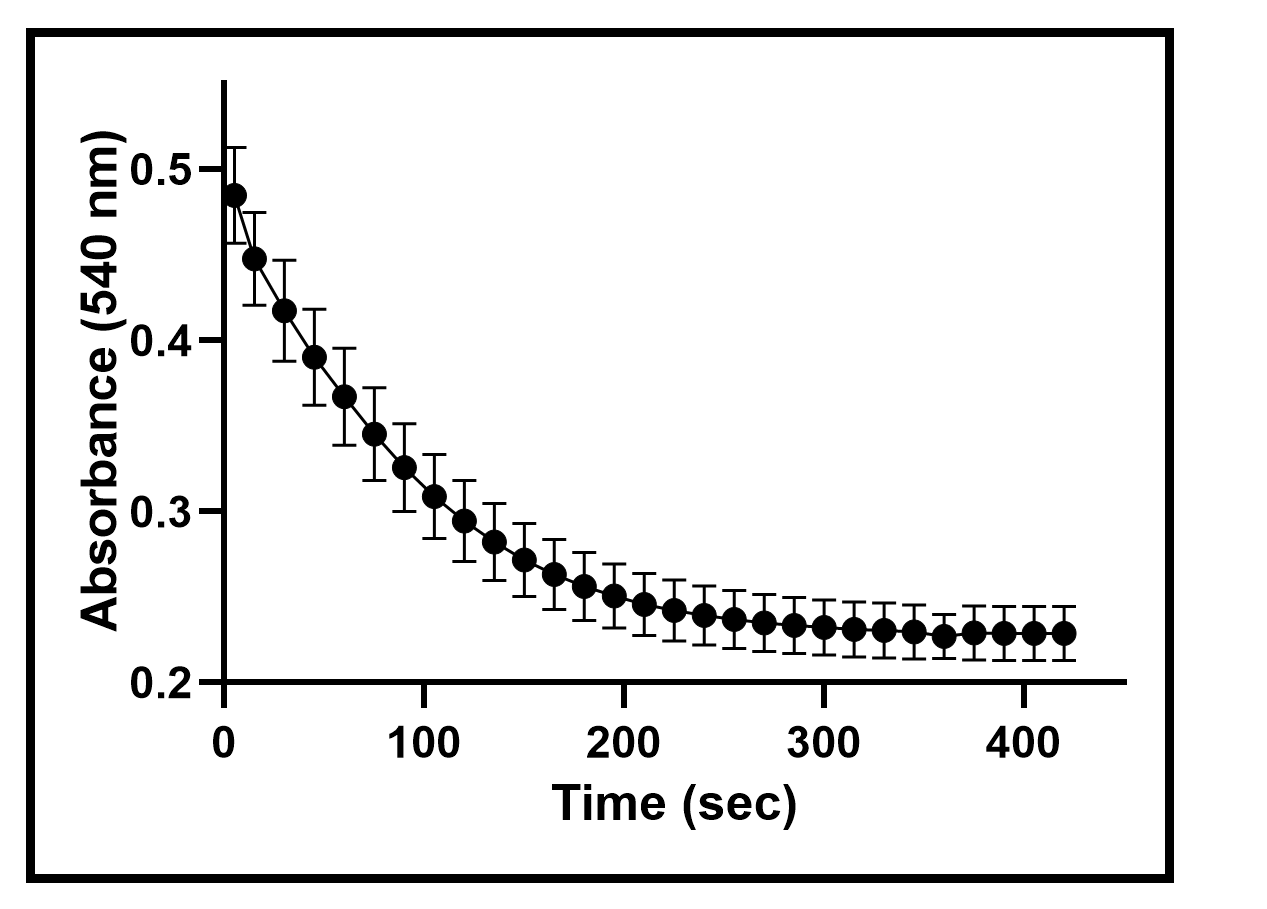


**Figure S2**. Methylcob(III)alamin:CoM methyl-transfer activity by MV10360. The reaction contained 0.5 mM methylcob(III)alamin, 50 mM phosphate buffer at pH 7.2, 5 mM CoM, 40 µg MV10360, and was performed anoxically under dim red light. Readings were taken every 15 s until completion. Demethylation of methylcob(III)alamin was measured by a decrease in absorbance at 540 nm. Error bars represent standard deviations (*n* = 3).


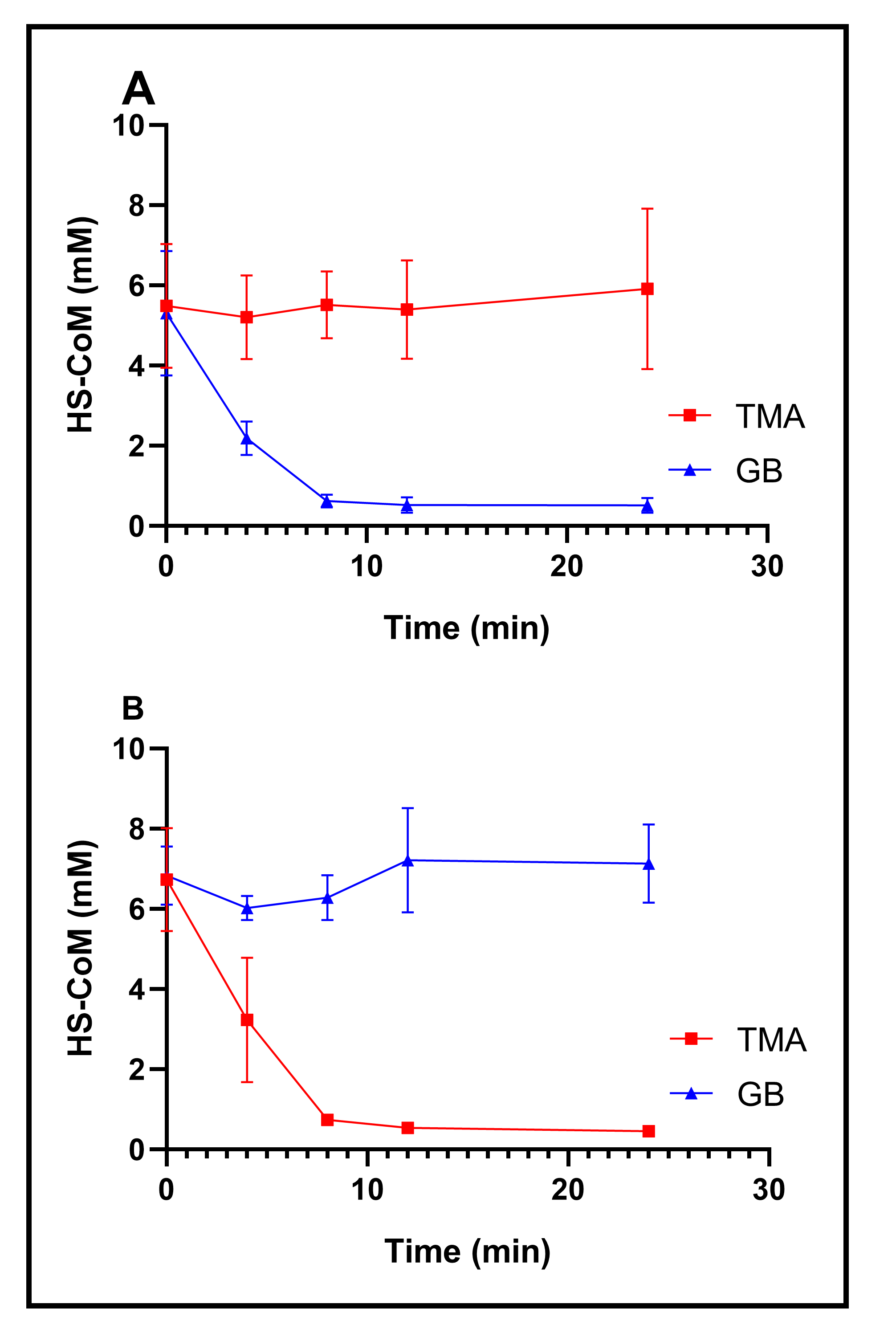


**Figure S3**. GB:CoM activity *in vitro* using crude extracts. Loss of the free thiol group on CoM was monitored using Ellman’s reagent at 412 nm. (**A**) Addition of GB to crude extracts from GB grown B1d cells resulted in a significant decrease in the amount of detectable HS-CoM (blue triangle), indicating an intact GB:CoM methyl transfer pathway. Detection of HS-CoM persisted when TMA was used as a methyl donor (red square), indicating a lack of a TMA:CoM methyl transfer pathway. (**B**) Addition of TMA to crude extracts from TMA grown B1d cells resulted in a significant decrease in the amount of detectable HS-CoM (red square), indicating an intact TMA:CoM methyl transfer pathway. Detection of HS-CoM persisted when GB was used as a methyl donor (blue triangle), suggesting a lack of a GB:CoM methyl transfer pathway. Error bars represent standard deviations (*n* = 3). No detectable decrease in HS-CoM was observed in any crude extracts when using methanol as the methyl donor, including from cells that were grown on methanol (data not shown).


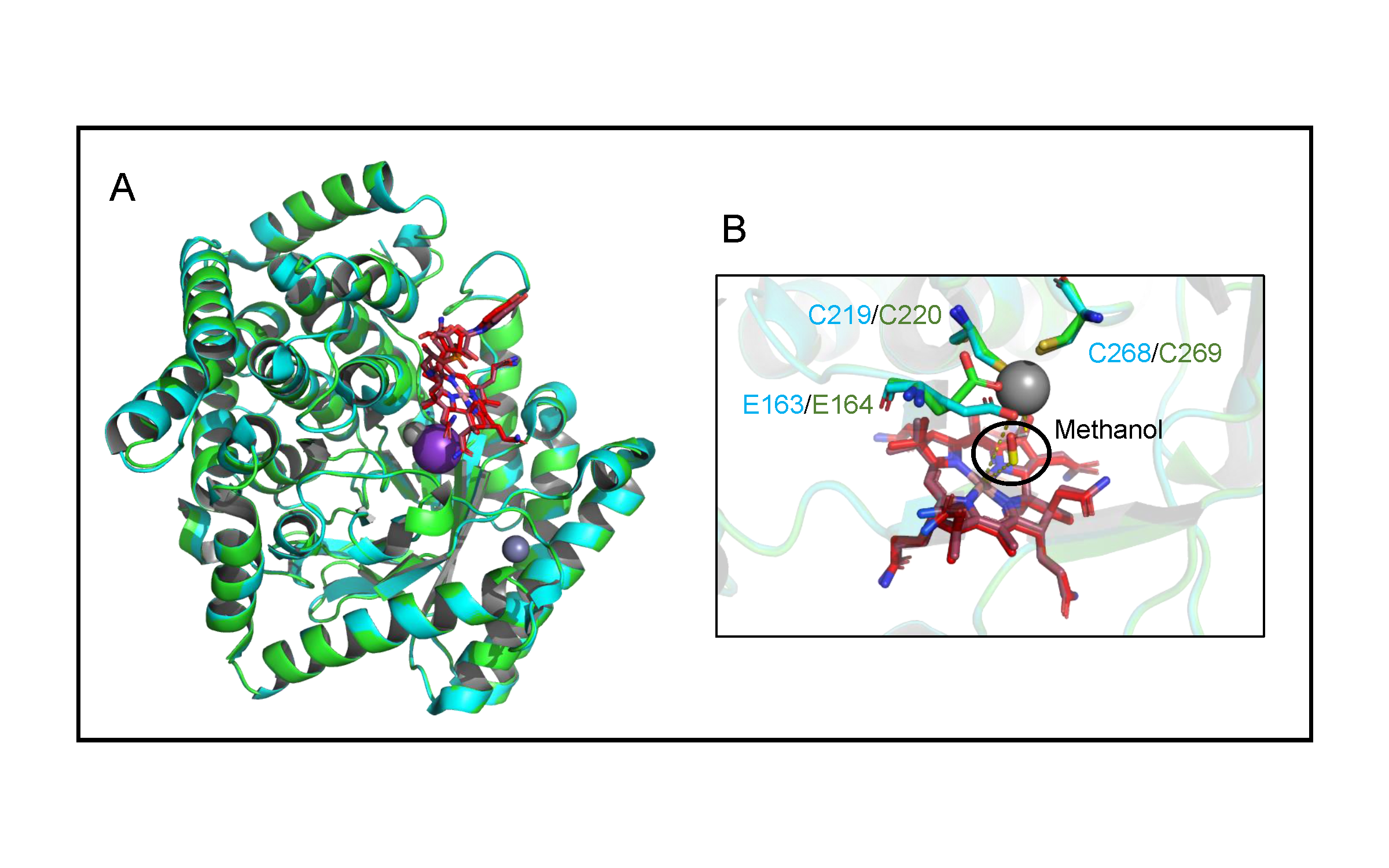


**Figure S4**. Predicted structural model of MV10350 compared to known MtaB from *Methanosarcina barkeri* Fusaro. (**A**) The global monomeric structure of MtaB (green) and predicted structure of MV10350 (cyan) are aligned. MtaB is bound with two zinc ions (gray), a predicted potassium ion (purple), and the cognate corrinoid ligand of MtaBC complex (red), while MV10350 is bound to zinc (grey) and cobalamin (tv-red). (**B**) The active site residues for zinc-binding are shown for MtaB (green) and MV10350 (cyan) relative to the cobalamin ligand (red, MtaB; tv-red, MV10350). Molecular docking of MV10350 for methanol using AutoDockVina (Trott and Olson, 2010) is highlighted with a black circle.

**CCATGGAACATCATCACCACCATCACGAAAACCTGTACTTCCAAGGCTACCGGTACCGCGGACTGCAGTTTAAA**ATGAATATGAAGGAGCGTCTGCTGAAGGCGCTGAAAGGCGAAGAAGTTGACAAAGTTCCGGTTTGCACCGTTACCCAGAGCGGCATCGTTGAGCTGATGGACAAGACCGGTGCGAGCTGGCCGGAAGCGCACAGCGACGCGAAAATGATGGCGGATCTGGCGTACGCGAGCTATGCGGAGTGCGGTCTGGAAGGCGTGCGTGCGCCGTACTGCCTGACCGTTCTGGCGGAGGCGATGGGTTGCACCATTAACATGGGCACCAAGAACCGTCAGCCGAGCGTTACCGATCACCCGTATCCGAAAGGTGTGGACGATCTGGCGATGCCGGAGGACCTGCTGAGCCAAGGCCGTATCCCGGTGGTTATGGAAGCGCTGGGTATTCTGCGTGAGAAGTGCGGCGATGAAGTGCCGGTTATCGCGGGTATGGAAGGTCCGGTTACCCTGGCGAGCGACCTGGCGAGCGTGAAGAAATTCATGAAATGGAGCATCAAGAAACCGGAGGATTTCCAGACCATTCTGGACTTTGCGTGCGATGCGTGCATCGAATATGCGAACGCGATGCTGGCGGCGGGTGCGGACGTGATTAGCGTTCCGGACCCGGTGGCGAGCCCGGACCTGATGGCGCCGGATGTGTTCGATAAGATTCTGAAACCGGTTCTGCAACGTTTTGCGGACGGTGTGAACGGCCCGATGATCCTGCACGTGTGCGGCGATGTTACCGCGATCATTGAGATGATGGCGGACTGCCACTTCGAAAGCATCAGCATTGAGGAAAAGGTTAAAGATCTGAAGGGTGCGAAGGCGAAAGTGGGCGACAAATGCACCATCTGCGGTAACGTGAGCAGCCCGTTTGTTCTGCTGGCGGGTGATGAAGCGGCGGTTAAAGCGGCGGCGAAACAGGCGCTGGATGATGGTATTGATGTGCTGGCGCCGGGTTGCGGCATCGCGCCGGATACCCCGGTTGCGAATCTGAAGGCGATGGTTGAAGCGCGTGATGACTACTATGCGTAA**CACGTGGGATCC**

**Figure S5**. Gene sequence of the optimized gene encoding MV10360 from GenScript. The bolded areas are the flanking regions that make up the multiple restriction site that was generated following removal of MV10360 from pET28_MV10360_Opt using DraI and PmlI to generate pETAC17a.


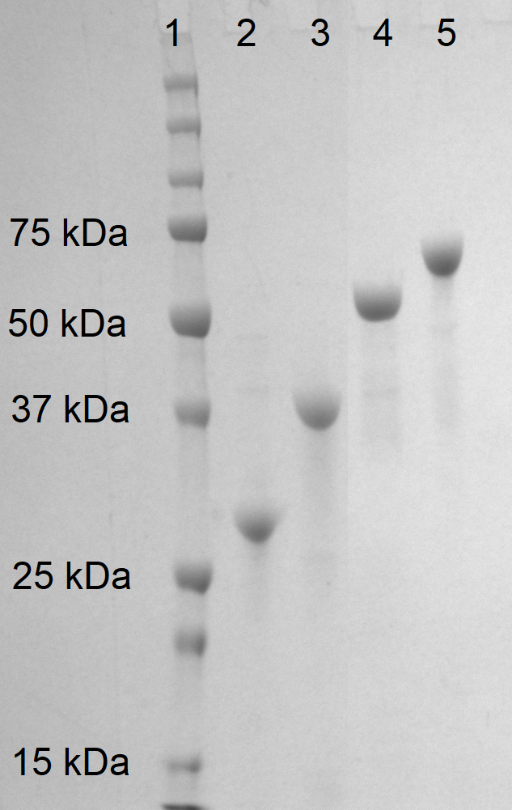


**Figure S6**. 12% acrylamide SDS-PAGE followed by coomassie blue staining of purified recombinant proteins used to reconstitute the GB:CoM methyl transfer pathway. Lane 1 is the molecular weight ladder. Lane 2 is MV8465 at ~28 kDa. Lane 3 is MV10360 at ~37 kDa. Lane 4 is MV8460 at ~53 kDa. Lane 5 is MV10335 at ~59 kDa. Lanes 2-5 each contained approximately 3 µg of protein. The image was digitally modified (seen between lanes 3 and 4) to remove a lane that consisted of a replicate of MV10360.

**Table S1**. Identification and location of gene products in this study.

| Gene Product | Locus Tag | Contig |
| --- | --- | --- |
| MV0375 | FKV42_00375 | paired contig 4 |
| MV0380 | FKV42_00380 | paired contig 4 |
| MV1575 | FKV42_01575 | paired contig 1 |
| MV1585 | FKV42_01585 | paired contig 1 |
| MV1595 | FKV42_01595 | paired contig 1 |
| MV1600 | FKV42_01600 | paired contig 1 |
| MV1615 | FKV42_01615 | paired contig 1 |
| MV1620 | FKV42_01620 | paired contig 1 |
| MV1650 | FKV42_01650 | paired contig 1 |
| MV1680 | FKV42_01680 | paired contig 1 |
| MV1685 | FKV42_01685 | paired contig 1 |
| MV1695 | FKV42_01695 | paired contig 1 |
| MV1720 | FKV42_01720 | paired contig 1 |
| MV1770 | FKV42_01770 | paired contig 1 |
| MV2245 | FKV42_02245 | paired contig 24 |
| MV8460 | FKV42_08460 | joined contig 2 |
| MV8465 | FKV42_08465 | joined contig 2 |
| MV10335 | FKV42_10335 | joined contig 2 |
| MV10345 | FKV42_10345 | joined contig 2 |
| MV10350 | FKV42_10350 | joined contig 2 |
| MV10360 | FKV42_10360 | joined contig 2 |

**Table S2**. Primers and plasmids used in this study.

| Primer name | Plasmid | Sequence | Product |
| --- | --- | --- | --- |
| MV8460 F | pASK-IBA43plus | ATGCCCGCGGAATGATACCAAAATTCGATG | pASK_MV8460 |
| MV8460 R | pASK-IBA43plus | ATCCCTCGAGTTTTTTCAATTCCGCAAACC |  |
| MV8465 + AsiSI F | pDL03c | GGCACTCGAGTAGGTGACCAGTCCCAAAATGATTTTAATAAATTAAGGAGCGATCGCATATGGTTACACAGGATGAAATTAATTC | pDLAC03_MV8465 |
| MV8465 + Tev R | pDL03c | CGCTTGGAAGTACAGGTTTTCACATTCACCTGCAGCTGCC |  |
| Tev + His R | pDL03c | GCATCCGCGGTCATTAGTGGTGGTGGTGGTGGTGCGCTTGGAAGTA  CAGGTTTTC |  |
| MV10335 F | pASK-IBA43plus | ATGCCCGCGGAATGAAAATTGGAGTTGCAATC | pASK_MV10335 |
| MV10335 R | pASK-IBA43plus | ATGCCTCGAGAGCTTCCTGTTCCATAACC |  |
| MV10360 F | pETAC17a | ATGCCCGCGGAATGAACATGAAAGAAAGATTACTC | pETAC_MV10360 |
| MV10360 R | pETAC17a | ATGCCTCGAGTTATTATGCGTAGTAATCGTCTC |  |
| pETAC17 Seq F | pETAC17a | ATGCGTCCGGCGTAGAG |  |
| pETAC17 Seq R | pETAC17a | GTTATTGCTCAGCGGTGG |  |
| pDL05c Seq F | pDL05c | CACTAGTGATCTAGATGCATG |  |
| pDL05cSeq R | pDL05c | CGACGTTGTAAAACGACGG |  |
|  | pDLAC03 + pDL05c |  | pDLAC05_MV8465 |

**References**

Hagemeier, C.H., Krer, M., Thauer, R.K., Warkentin, E. and Ermler, U. (2006). Insight into the mechanism of biological methanol activation based on the crystal structure of the methanol-cobalamin methyltransferase complex. Proc. Natl. Acad. Sci. U.S.A. 103, 18917-18922.

Tallant, T.C. and Krzycki, J.A. (1997). Methylthiol:coenzyme M methyltransferase from *Methanosarcina barkeri*, an enzyme of methanogenesis from dimethylsulfide and methylmercaptopropionate. J. Bacteriol. 179, 6902-6911.

Trott, O. and Olson, A.J. (2010). AutoDock Vina: improving the speed and accuracy of docking with a new scoring function, efficient optimization, and multithreading. J. Comput. Chem. 31, 455-461.

Zhang, Y. (2008). I-TASSER server for protein 3D structure prediction. BMC Bioinformatics. 9, 40.
